# Editorial: Examining upstream to understand downstream: use of telehealth and other health equity measures for addressing health disparities

**DOI:** 10.3389/fpubh.2024.1529825

**Published:** 2024-12-09

**Authors:** Gulzar H. Shah, Kristie Cason Waterfield, Tran Nguyen, Sripriya Rajamani

**Affiliations:** ^1^Department of Health Policy and Community Health, Jiann-Ping Hsu College of Public Health, Georgia Southern University, Statesboro, GA, United States; ^2^Department of Health Management, Economics, & Policy, Institute of Public and Preventive Health, Augusta University, Augusta, GA, United States; ^3^Population Health and Systems Cooperative, School of Nursing, University of Minnesota, Minneapolis, MN, United States

**Keywords:** telehealth, health equity (MeSH), Social Determinants of Health, digital divide, population health, culturally appropriate care

## Introduction

Modern public health and healthcare focus on superior, equitable, well-coordinated access to services to improve population health (1). The present-day healthcare systems strive to be among the early adopters of several paradigm shifts (2). The shifts in healthcare include provider-driven to patient-centric care and a shift from siloed facility care to superior care coordination across specialties and healthcare settings. Big data, artificial intelligence (AI)-driven predictive analytics, and precision medicine support healthcare providers in this transition. Public health agencies aim to align with the Public Health 3.0 approach comprising collaborative policy referred to as Health in all Policies and promoting equity in Social Determinants of Health (SDoH) “upstream” to eliminate avoidable health disparities “downstream” (3, 4). Perhaps the most significant of all of these paradigm shifts is a shift in the goal of healthcare from profit-driven to population health outcomes-driven, striving to break the silos between healthcare and public health organizations (5, 6). The COVID-19 pandemic heightened the need to address the structural inequities in SDoH, such as access to technology, blue-collar work, poor housing, lack of support services, and geographical challenges to access to care (1).

This Research Topic titled “*Examining upstream to understand downstream: use of telehealth and other health equity measures for addressing health disparities*” showcases articles covering telehealth and other health equity measures as ways to improve health equity. This Research Topic highlights the role of telehealth as an equalizer for health equity. The aim is to highlight the successes and challenges of telehealth as a health equity tool for underserved and marginalized populations facing a shortage of care providers, particularly in various specialty areas. The practice-relevant and policy-relevant research evidence produced in this Research Topic can guide interventions aiming to advance health outcomes while enabling fair and accessible health care.

## Research on telehealth implementation for underserved populations

Williams et al. make a convincing case for addressing the healthcare challenges of incarcerated populations because of biases in the justice system and socio-cultural deprivations. The need for telehealth carceral facilities became critical during the COVID-19 pandemic to protect the justice-impacted individuals. This paper discussed upstream factors limiting public health services and medical care access in correctional facilities due to numerous challenges, including policy gaps, flaws in the design of the telehealth technologies for correctional facilities, ethical and legal challenges, security and privacy issues for the service providers, and operational difficulties. The authors present a pathway supported by telehealth for increased healthcare access, early screening and diagnosis, and healthcare-provider interaction for community connectivity outside correctional facilities. They also share a model for improved telehealth access for the “justice-impacted” populations.

## Research on programs/initiatives for disparities reduction through telemedicine

The methodological rigorous research by Kobashi et al. involved the use of machine learning and historical cohort design to examine hospital visit behaviors of patients after receiving physicians' telehealth-supported advice. The study showed that physician advice shaped patients' adherence to physician-recommended hospital visits, but differences existed based on patient's characteristics. This study offered insights into the need for optimizing telehealth applications and equitable health resource allocation upstream to reduce downstream disparities in adherence to medical advice and access to care. The authors also highlight disadvantages concerning telehealth use for population subgroups such as older adults and those with behavioral health issues.

## Research on health policy and telehealth

Policy reforms from the Centers for Medicare and Medicaid Services (CMS) may influence telehealth adoption and access for vulnerable populations, particularly CMS 1135 and the CHRONIC Care Act (7, 8). These policies increase telehealth coverage for Traditional Medicare enrollees and enhance access to telehealth for managing chronic conditions. Wang et al. examined the differences in the availability and use of telehealth services among Medicare enrollees based on their status regarding Alzheimer's disease and related dementias (ADRD), as well as their enrollment in Medicare Advantage (MA) vs. Traditional Medicare (TM) during the COVID-19 pandemic. The authors found no significant differences in the availability or utilization of telehealth services between Medicare beneficiaries with ADRD and those without. Although telehealth services were more readily available to Medicare enrollees prior to the pandemic, due to heightened demand for these services, this was not the case during the pandemic.

## Research on equity and digital divide in telehealth

Aldekhyyel et al.'s data-driven research focuses on addressing health equity upstream by understanding health disparities to make a case for investments in telehealth. They examined behavioral intentions and e-health literacy shaping the potential use of telemedicine in the post-COVID-19 pandemic, spreading their positive impact on the adoption of telemedicine. Post-COVID, addressing the rural-urban digital health divide is even more critical to utilizing telehealth as an equalizer to address health inequities. Improving digital health literacy will address the barriers to telemedicine adoption, including complex user interfaces and limited digital skills, and enhance telemedicine participation among older adults and persons with chronic comorbidities.

Hernandez et al. also examined the digital divide in telehealth screening for disadvantaged population subgroups. The authors reason that ensuring inclusive access to healthcare and equitable digital screening contributes to health equity. The authors make a case for collaborations among healthcare and community-based organizations and federal agencies such as CMS charged with improved healthcare for disadvantaged populations to incentivize healthcare service providers to ensure inclusion in digital health screening. The authors recommend ensuring inclusive access and equity-centered digital screening through culturally appropriate tools, adapting to patients' digital literacy, and addressing limited internet access.

## Research on health equity beyond telehealth

The characteristics of neighborhoods, towns, and cities can significantly influence the health and wellbeing of their residents through physical, social, and economic factors and impact SDOH. Just before the COVID-19 pandemic, Ocaña-Ortiz et al., from the Valencian Community, Spain, used the Place Standard Tool (PST) to assess how the community perceived their municipality regarding these aspects, exploring the differences between rural and urban contexts. The PST facilitates discussions about SDOH, organized into 14 themes, and serves as a foundation for local health interventions. Their findings validated that PST is valuable for promoting local health due to its versatility and action-oriented approach.

To address the disparities in healthcare that negatively affect minority ethnic populations in England, Obita et al. conducted a study examining childhood obesity and related health issues from the perspectives of parents within Black, Asian, and Minority Ethnic (BAME) communities in Northeast England. The authors discovered that the views of these communities on childhood obesity prevention do not align with the preventative services offered by the healthcare system. They emphasized the need for community and family-oriented approaches to prevent obesity, particularly through lifestyle interventions. The study underlines the need for culturally appropriate strategies to prevent obesity and its associated comorbidities in minority ethnic communities living in high-income countries.

## Conclusions

Continuing to examine the SDoH “upstream” factors that influence health disparities “downstream” is crucial because it allows for a more comprehensive understanding of the root causes of health inequities (1). This allows for more effective and lasting solutions to reduce health disparities across underserved and vulnerable populations. Researchers, policymakers, and practitioners can utilize telehealth to create a more equitable healthcare system by increasing access to early screening and diagnosis, optimizing telehealth resource allocation, examining patient behavioral intentions, measuring e-health literacy, and incentivizing healthcare service providers to deliver more inclusive digital health screenings. Collaboration across sectors and stakeholders is vital and includes data gathering and sharing SDOH “upstream” data, community involvement in technology development and deployment, flexible funding for social needs, and supporting backbone organizations (9).
